# Supplementary material for: Co-benefits of protecting mangroves for biodiversity conservation and carbon storage
Source: Nat Commun. 2021 Jun 23;12:3875. doi: 10.1038/s41467-021-24207-4 (PMC8222281; doi:10.1038/s41467-021-24207-4)
Supplement: Supplementary file 1 — Supplemetary Information [file 41467_2021_24207_MOESM1_ESM.pdf]

Supplementary Information

Co-benefits of protecting mangroves for biodiversity conservation and carbon storage

Md Mizanur Rahman[1, 2,3*], Martin Zimmer[4] , Imran Ahmed[5], Daniel Donato[6], Mamoru Kanzaki[3], Ming Xu[1,2*]

[1]Henan Key Laboratory of Earth System Observation and Modeling, Henan University, Kaifeng 475004, China

[2]College of Environment and Planning, Henan University, Kaifeng 475004, China

[3]Graduate School of Agriculture, Kyoto University, Kitashirakawa Oiwake, Sakyo-ku, Kyoto, 606-8502 Japan

[4]Leibniz Centre for Tropical Marine Research & University of Bremen, Bremen, Germany & IUCN-SSC Mangrove Specialist Group

[5]Bangladesh Forest Department, Bon Bhaban, Plot No- E-8, B-2, Agargaon, Sher-e-Bangla Nagar, Dhaka, 1207 Bangladesh

[6]School of Environmental and Forest Sciences, University of Washington, Seattle, WA, 98195, USA.

*Correspondence to: mingxu@henu.edu.cn, mizanfwt04@gmail.com

Supplementary figures and tables

Supplementary figures

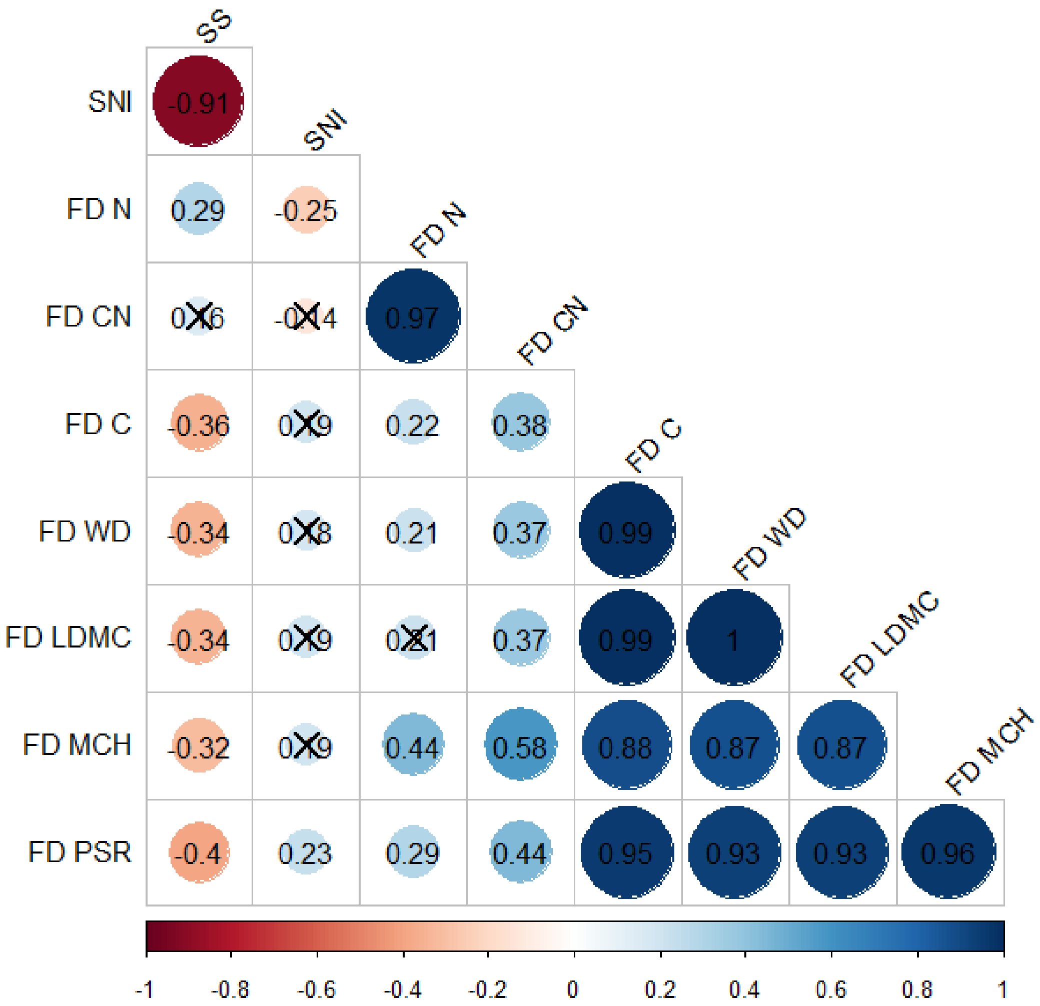

Supplementary Fig. 1: Pair wise correlation between species richness and functional diversity of different traits. Block with cross indicating insignificant relationship between variables (P > 0.05). SS: Sediment salinity, SNI: Sediment nutrient index, FC: Functional diversity, MCH: Maximum canopy height, WD: Wood density, PSR: Leaf photosynthesis rate, C: Leaf litter carbon content, N: Leaf litter nitrogen content, C: N: leaf litter carbon to nitrogen ratio, LDMC: Leaf litter dry matter Content.

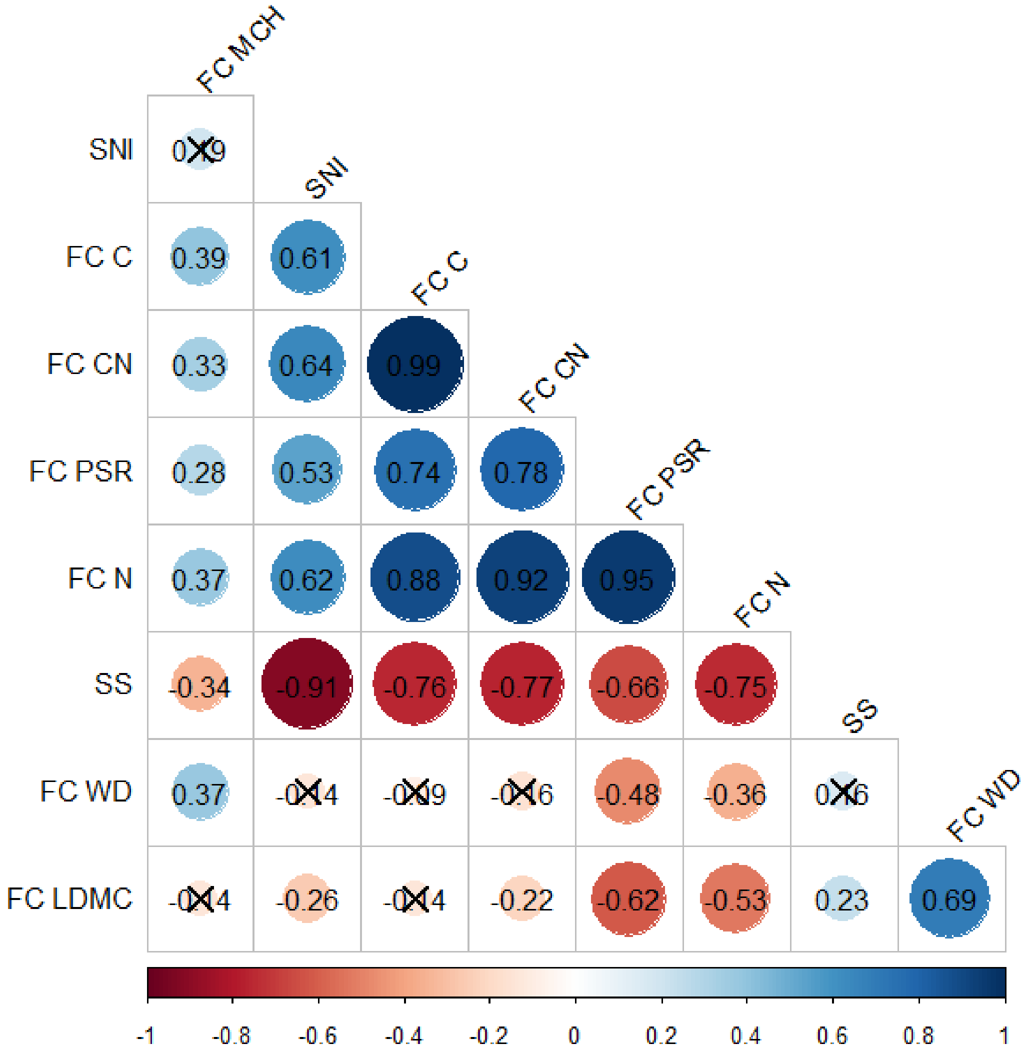

Supplementary Fig. 2: Pair wise correlation between species richness and functional composition of different traits. Block with cross indicating insignificant relationship between variables (P > 0.05). SS: Sediment salinity, SNI: Sediment nutrient index, FD: Functional composition, MCH: Maximum canopy height, WD: Wood density, PSR: Leaf photosynthesis rate, C: Leaf litter carbon content, N: Leaf litter nitrogen content, C: N: leaf litter carbon to nitrogen ratio, LDMC: Leaf litter dry matter Content.

Supplementary Tables

Supplementary Table 1. Summary of species richness, soil properties and blue carbon storage in the Sundarbans Reserved Forest based on the disturbance's free plots of the 2009-2010 carbon inventory.

Statistics	Species richness (number plot^{-1})	Sediment nutrient index	Sediment salinity (mSm^{-1})	Aboveground plant biomass carbon (Mgha^{-1})	Belowground plant biomass carbon (Mgha^{-1})	Sediment organic carbon (Mgha^{-1})	Total ecosystem carbon (Mgha^{-1})
N	90	90	90	90	90	90	90
Mean	3.60	4.56	9.10	75.32	39.53	119.40	276.70
Median	3.00	4.21	9.34	68.09	36.68	115.90	247.30
Min	2.00	2.62	4.56	10.13	6.09	53.46	104.60
Max	8.00	7.30	14.46	263.10	134.70	304.40	598.30
Range	6.00	4.67	9.90	253.00	128.60	250.90	493.70
1st Quartile	3.00	3.79	7.61	33.39	19.42	90.34	179.50
3rd Quartile	4.00	5.30	10.33	104.50	54.36	141.20	332.90

Standard Deviation	1.44	1.04	2.03	51.55	25.69	41.73	122.90
Coefficient of Variation	0.40	0.23	0.22	0.68	0.65	0.35	0.44

Supplementary Table 2. Species wise trait that were used in this study in the Sundarbans Reserved Forest.

Species	RA	[1]WD gcm^{-3}	[2]PSR $\mu molm^{-2}s^{-1}$	[3]Litter C %	[3]Litter N %	[3]Litter C: N	[3]LDMC mg/gm	[4]MCH m
Heritiera fomes	52.95	1.07	13.21	55.25	1.43	38.7	0.73	38.5
Excoecaria agallocha	41.05	0.45	7.02	43.65	1.32	35.73	0.22	31
Xylocarpus mekongensis	2.42	0.73	5.36	47.54	2.97	16.07	0.45	26.4
Bruguiera sexangula	1.41	0.86	5.89	54.3	1.22	44.73	0.47	21
Avicennia officinalis	0.71	0.67	8.39	47.45	1.94	24.47	0.43	31.3
Amoora cuculata	0.42	0.66	6.41	44.95	1.86	29.22	0.41	21.5
Xylocarpus granatum	0.42	0.7	3.47	45.56	2.34	19.43	0.37	18
Intsia bijuga	0.2	0.8	6.41	44.95	1.86	29.22	0.41	10.6
Sonneratia apetala	0.07	0.56	6.41	43.54	1.84	23.73	0.26	29.9
Sonneratia caseolaris	0.07	0.5	6.41	51.79	2.63	19.73	0.31	10
Cynometra ramiflora	0.06	0.9	6.41	44.95	1.86	29.22	0.41	13.9
Aegiceras corniculatum	0.05	0.64	6.75	54.88	1.11	49.67	0.63	9.8
Unknown species	0.05	0.73	6.41	44.95	1.86	29.22	0.41	16.5
E. indica	0.04	0.65	6.41	44.95	1.86	29.22	0.41	15
Pongamia pinnata	0.04	0.67	6.41	44.95	1.86	29.22	0.41	18.5
Rhizophora mucronata	0.04	1.02	5.72	52.15	0.92	56.93	0.44	16.5
Hibiscus tiliaceus	0.01	0.6	6.41	44.95	1.86	29.22	0.41	7.8
Lumnitzera racemosa	0	0.73	6.41	43.72	1.96	22.43	0.23	16

[1]Yakub et al.[55] , [2]Nandy et al. [56], [3]Chanda et al. [36], [4]Sundarbans Forest Inventory 1997

RA: Relative abundance. MCH: Maximum tree height, WD: Wood Density, PSR: Leaf photosynthesis rate, C: Litter Carbon, N: Litter Nitrogen, C: N: Litter Carbon to Nitrogen ratio, LDMC: Leaf litter Dry Matter Content

Supplementary Table 3. Result of variance inflation factors test for checking multicollinearity among functional diversity of different traits along with species richness and Shannon diversity. Multiple linear regression (Total ecosystem carbon used as response variable) was used where regression started by including all the covariates and gradually removes the variable with highest variance inflation factor until achieving the threshold value. Covariates with a variance inflation factor below 10 (threshold value) do not imply multicollinearity.

Steps	Species richness	Shannon diversity	FD LDMC	FD MCH	FD WD	FD PSR	FD C	FD N	FD C:N
1.00	2.09	6.07	280.10	25.30	667.30	47.07	216.84	41.22	48.89
2.00	2.09	5.89	46.62	23.81	NA	43.31	66.44	32.16	38.39
3.00	2.09	5.80	9.66	21.98	NA	34.17	NA	32.02	38.15
4.00	1.94	5.75	8.96	21.70	NA	34.17	NA	2.09	NA
5.00	1.94	5.12	5.39	8.98	NA	NA	NA	1.70	NA

FD: Functional diversity, MCH: Maximum canopy height, WD: Wood density, PSR: Leaf photosynthesis rate, C: Leaf litter carbon content, N: Leaf litter nitrogen content, C: N: leaf litter carbon to nitrogen ratio, LDMC: Leaf litter dry matter Content

Supplementary Table 4. Result of variance inflation factors test for checking multicollinearity among functional composition of different traits along with species richness and Shannon diversity. Multiple linear regression (Total ecosystem carbon used as response variable) was used where regression started by including all the covariates and gradually removes the variable with highest variance inflation factor until achieving the threshold value. Covariates with a variance inflation factor below 10 (threshold value) do not imply multicollinearity.

Steps	Species richness	Shannon diversity	FC WD	FC MCH	FC PSR	FC C	FC N	FC C:N	FC LDMC
1	2.03	6.84	741.05	54.04	234.57	206.55	34.06	47.14	1053.8
2	1.96	3.41	166.22	53.81	189.39	198.26	34.02	34.04	NA
3	1.91	1.94	42.46	48.47	166.68	NA	28.04	26.2	NA
4	1.61	1.84	6.53	6.45	NA	NA	24.33	25.99	NA
5	1.52	1.84	4.96	6.42	NA	NA	2.72	NA	NA

FC: Functional diversity, MCH: Maximum canopy height, WD: Wood density, PSR: Leaf photosynthesis rate, C: Leaf litter carbon content, N: Leaf litter nitrogen content, C: N: leaf litter carbon to nitrogen ratio, LDMC: Leaf litter dry matter Content

Supplementary Table 5: Results fit statistics of all tested structural equation models for predicting different carbon pools in combination of biotic and abiotic predictors. Model with insignificant Chi value ($\chi2$, P>0.05, bold) square along with comparative fit index close to one (CFI) and standardized root mean square residual close to zero (SRMR), indicates no significant deviation of model from observed dataset and thus indicates good fit.

Response variables	Predictors		Fit indices						Remarks
	Biotic	Abiotic	R^2	χ^2	df	p-value	CFI	SRMR	
AGPC	SR, FD LDMC, FD MCH, FD N, FC MCH, FC WD, FC N	SS, SNI	0.69	177.163	9	0.000	0.823	0.065	Rejected
AGPC	SR, FD LDMC, FD MCH, FC MCH, FC WD, FC N	SS, SNI	0.69	101.434	6	0.000	0.88	0.038	Rejected
AGPC	SR, FD LDMC, FC MCH, FC WD, FC N	SS, SNI	0.68	8.183	3	0.042	0.991	0.026	Rejected
AGPC	SR, FD LDMC, FC MCH, FC WD, FC N	SS	0.69	6.419	3	0.093	0.991	0.036	Accepted
AGPC	H, FD LDMC, FD MCH, FD N, FC MCH, FC WD, FC N	SS, SNI	0.66	193.385	9	0.000	0.825	0.089	Rejected
AGPC	H, FD LDMC, FD MCH, FC MCH, FC WD, FC N	SS, SNI	0.66	112.935	6	0.000	0.883	0.066	Rejected
AGPC	H, FD LDMC, FC MCH, FC WD, FC N	SS, SNI	0.66	54.737	3	0.000	0.923	0.072	Rejected
AGPC	H, FD LDMC, FC MCH, FC WD, FC N	SS	0.66	55.387	3	0.000	0.895	0.082	Rejected
BGPC	SR, FD LDMC, FD MCH, FD N, FC MCH, FC WD, FC N	SS, SNI	0.68	177.163	9	0.000	0.823	0.064	Rejected
BGPC	SR, FD LDMC, FD MCH, FC MCH, FC WD, FC N	SS, SNI	0.68	101.434	6	0.000	0.88	0.038	Rejected
BGPC	SR, FD LDMC, FC MCH, FC WD, FC N	SS, SNI	0.68	8.183	3	0.042	0.991	0.026	Rejected
BGPC	SR, FD LDMC, FC MCH, FC WD, FC N	SS	0.69	6.419	3	0.093	0.991	0.036	Accepted

BGPC	H, FD LDMC, FD MCH, FD N, FC MCH, FC WD, FC N	SS, SNI	0.66	193.385	9	0.000	0.824	0.089	Rejected
BGPC	H, FD LDMC, FD MCH, FC MCH, FC WD, FC N	SS, SNI	0.66	112.935	6	0.000	0.883	0.066	Rejected
BGPC	H, FD LDMC, FC MCH, FC WD, FC N	SS, SNI	0.66	54.737	3	0.000	0.923	0.071	Rejected
BGPC	H, FD LDMC, FC MCH, FC WD, FC N	SS	0.66	55.387	3	0.000	0.895	0.082	Rejected
SOC	SR, FD LDMC, FD MCH, FD N, FC MCH, FC WD, FC N	SS, SNI	0.34	177.163	9	0.000	0.81	0.065	Rejected
SOC	SR, FD LDMC, FD MCH, FC MCH, FC WD, FC N	SS, SNI	0.32	101.434	6	0.000	0.869	0.037	Rejected
SOC	SR, FD LDMC, FC MCH, FC WD, FC N	SS, SNI	0.32	8.183	3	0.042	0.989	0.026	Rejected
SOC	SR, FD LDMC, FC MCH, FC WD, FC N	SS	0.26	6.419	3	0.093	0.989	0.032	Accepted
SOC	H, FD LDMC, FD MCH, FD N, FC MCH, FC WD, FC N	SS, SNI	0.30	193.385	9	0.000	0.813	0.09	Rejected
SOC	H, FD LDMC, FD MCH, FC MCH, FC WD, FC N	SS, SNI	0.30	112.935	6	0.000	0.874	0.065	Rejected
SOC	H, FD LDMC, FC MCH, FC WD, FC N	SS, SNI	0.30	54.737	3	0.000	0.915	0.07	Rejected
SOC	H, FD LDMC, FC MCH, FC WD, FC N	SS	0.20	55.387	3	0.000	0.875	0.082	Rejected
TEC	SR, FD LDMC, FD MCH, FD N, FC MCH, FC WD, FC N	SS, SNI	0.61	177.163	9	0.000	0.82	0.063	Rejected
TEC	SR, FD LDMC, FD MCH, FC MCH, FC WD, FC N	SS, SNI	0.61	101.434	6	0.000	0.878	0.037	Rejected
TEC	SR, FD LDMC, FC MCH, FC WD, FC N	SS, SNI	0.61	8.183	3	0.042	0.99	0.026	Rejected
TEC	SR, FD LDMC, FC MCH, FC WD, FC N	SS	0.61	6.419	3	0.093	0.99	0.035	Accepted
TEC	H, FD LDMC, FD MCH, FD N, FC MCH, FC WD, FC N	SS, SNI	0.58	193.385	9	0.000	0.821	0.091	Rejected
TEC	H, FD LDMC, FD MCH, FC MCH, FC WD, FC N	SS, SNI	0.58	112.935	6	0.000	0.881	0.068	Rejected

TEC	H, FD LDMC, FC MCH, FC WD, FC N	SS, SNI	0.57	54.737	3	0.000	0.921	0.072	Rejected
TEC	H, FD LDMC, FC MCH, FC WD, FC N	SS	0.56	55.387	3	0.000	0.89	0.083	Rejected

AGPC: Aboveground plant biomass carbon, BGPC: Belowground Plant biomass carbon, SOC: Sediment Organic Carbon, TEC: Total Ecosystem Carbon, SR: Species richness, H: Shannon Diversity Index, MCH: Maximum Canopy Height, WD: Wood Density, N: Leaf Litter Nitrogen, LDMC: Leaf litter Dry Matter Content, FC: Functional Composition, FD: Functional Diversity, SS: Sediment salinity, SNI: Sediment nutrient index, R^2: Coefficient of determination, χ^2: Chi square, DF: Degree of freedom, CFI: Comparative fit index, SRMR: Standardized Root Mean Square Residual.

Supplementary Table 6. Result of variance inflation factor test for checking multicollinearity using multiple linear regression among the covariates in the accepted structural equation models (a similar variance inflation factors were found for all the covariates and for the different blue carbon storage). Covariates with a value below 10 do not imply multicollinearity.

Steps	Species richness	Functional diversity Leaf litter dry matter content	Functional composition Wood density	Functional composition maximum canopy height	Functional composition leaf litter nitrogen	Sediment salinity
1	1.52	1.21	4.88	6.15	2.81	2.63
