# Supplementary material for: Co-benefits of protecting mangroves for biodiversity conservation and carbon storage
Source: Nat Commun. 2021 Jun 23;12:3875. doi: 10.1038/s41467-021-24207-4 (PMC8222281; doi:10.1038/s41467-021-24207-4)
Supplement: Supplementary file 3 — Reporting Summary [file 41467_2021_24207_MOESM3_ESM.pdf]

nature research

Corresponding author(s): Ming Xu

Last updated by author(s): May 20, 2021

Reporting Summary

Nature Research wishes to improve the reproducibility of the work that we publish. This form provides structure for consistency and transparency in reporting. For further information on Nature Research policies, see our Editorial Policies and the Editorial Policy Checklist.

Statistics

For all statistical analyses, confirm that the following items are present in the figure legend, table legend, main text, or Methods section.

n/a	Confirmed	
☐	☒	The exact sample size (*n*) for each experimental group/condition, given as a discrete number and unit of measurement
☐	☒	A statement on whether measurements were taken from distinct samples or whether the same sample was measured repeatedly
☐	☒	The statistical test(s) used AND whether they are one- or two-sided<br>*Only common tests should be described solely by name; describe more complex techniques in the Methods section.*
☐	☒	A description of all covariates tested
☐	☒	A description of any assumptions or corrections, such as tests of normality and adjustment for multiple comparisons
☐	☒	A full description of the statistical parameters including central tendency (e.g. means) or other basic estimates (e.g. regression coefficient) AND variation (e.g. standard deviation) or associated estimates of uncertainty (e.g. confidence intervals)
☐	☒	For null hypothesis testing, the test statistic (e.g. *F*, *t*, *r*) with confidence intervals, effect sizes, degrees of freedom and *P* value noted<br>*Give P values as exact values whenever suitable.*
☒	☐	For Bayesian analysis, information on the choice of priors and Markov chain Monte Carlo settings
☒	☐	For hierarchical and complex designs, identification of the appropriate level for tests and full reporting of outcomes
☐	☒	Estimates of effect sizes (e.g. Cohen's *d*, Pearson's *r*), indicating how they were calculated

Our web collection on statistics for biologists contains articles on many of the points above.

Software and code

Policy information about availability of computer code

Data collection	No software was used.
Data analysis	For statistical analysis we used R version 3.6.3.. We used several R packages for data analyzing, statistical testing and visualization such as FD, rstatix,psych,car,plyr,lavaan,ggplot2,ggpmisc, ggpubr. We also used Microsoft excel and power point.

For manuscripts utilizing custom algorithms or software that are central to the research but not yet described in published literature, software must be made available to editors and reviewers. We strongly encourage code deposition in a community repository (e.g. GitHub). See the Nature Research guidelines for submitting code & software for further information.

Data

Policy information about availability of data

All manuscripts must include a data availability statement. This statement should provide the following information, where applicable:

- Accession codes, unique identifiers, or web links for publicly available datasets
- A list of figures that have associated raw data
- A description of any restrictions on data availability

The datasets analyzed during the current study are available from the corresponding authors on reasonable request.

Field-specific reporting

Please select the one below that is the best fit for your research. If you are not sure, read the appropriate sections before making your selection.

☐ Life sciences ☐ Behavioural & social sciences ☒ Ecological, evolutionary & environmental sciences

For a reference copy of the document with all sections, see nature.com/documents/nr-reporting-summary-flat.pdf

Ecological, evolutionary & environmental sciences study design

All studies must disclose on these points even when the disclosure is negative.

Study description

We explored the association of species richness, taxonomic diversity, functional diversity and functional composition with aboveground and belowground plant biomass carbon, sediment organic carbon and total ecosystem carbon storage after considering for the effect of sediment salinity, using a structural equation model. Field inventory data were by following standard protocol for mangrove carbon pools (live and dead standing trees and saplings, seedling, down woody debris, sediment organic carbon storage) assessment. Sediment salinity, sediment nutrients indices and plant traits were collected from publicly available data sources. According to structural equation models, sediment salinity, species diversity, functional diversity and functional composition together explain 69%, 69%, 27% and 61% of the variation in aboveground carbon, belowground carbon, sediment organic carbon and total ecosystem carbon storage, respectively. Our findings indicate that blue carbon storage in mangroves can be best sustained through compositionally and functionally diverse tree assemblages.

Research sample

Sediment samples were collected at one-meter depth with two depth range: at the midpoint of 0–30 cm (15-20 cm) and 30–100 cm (60-65 cm) depth intervals from each of the five subplots of 150 plots. For developing biomass conversion factor for vegetations (shrubs, herbs, palm, seedling, etc.) that surveyed by counting, we collect 30 sample for each species.

Sampling strategy

Following a systematic grid sampling process, 155 plots (consisting of five 10-m radius circular subplots) were selected at 4 min latitude intervals and 2 min longitude intervals. Of the 155 plots, five plots were under water.

Data collection

Two field groups were formed that led by Assistant Conservator of Forest (ACF). Each group consisted of one ACF Conservator, one Forest Ranger / Deputy Ranger, two foresters, two students, two staff and two armed guards. Usually, ACF and Forest Ranger/ Deputy Forest Ranger recorded the data while foresters and students were measured the inventory parameters.

Timing and spatial scale

From December 5, 2009, to April 30, 2010, field inventories were performed. Since heavy rainfall occurs during the monsoon season (June to September), the best time to visit the Sundarbans is during the post-monsoon (October–January) and pre-monsoon (February–May).

Data exclusions

Out of the150 surveyed plots, we used 90 (90 x 5 subplots) plots in our current study. The excluded 60 plots, had water bodies > 10 %, were completely underwater, or were severely disturbed by cyclone or fire.

Reproducibility

We followed bootstrap methods validating the coefficients of Structural Equation Models .

Randomization

Not applicable

Blinding

Not applicable

Did the study involve field work? ☒ Yes ☐ No

Field work, collection and transport

Field conditions

Sundarbans Reserved Forest is a mangrove forest. Soil condition was muddy in some plots and some are hard usual upper land. The mean annual maximum and minimum temperatures vary between 32 degree Celsius and 20 degree Celsius. Mean annual relative humidity varies from 77 to 80 %. The mean annual rainfall ranges between 1900 and 2500 mm.

Location

The study was carried out in the Sundarbans Reserved Forest, Bangladesh which lies between 21 degree 30 minute to 22 degree 30 minute N and 89 degree 00 minute to 89 degree 55 minute E. This forest covers a total area of 6,017 square kilometers, with trees covering 4,120 square kilometers and water-bodies covering 1,897 square kilometers.

Access & import/export

We used small boats to reach the nearest position to each plot through small river and canals in order to reduce walking distance in this dense forest. Some time we made path for easy access in dense shrubs and herbs. For safe arrival, we recorded the boat's waypoint (latitude and longitude) and put flagging tape on trees along the path leading to the plot.

Disturbance

Since we collected 5 cm soil cores as described in the method, the sampling method caused minimum damage relevant environments. We also harvested 30 individuals for developing biomass conversion factor for non tree vegetation which caused minor damage. For accessing to exact plot location, sometimes, we made path by cutting dense vegetation which were unavoidable and damage some non timber vegetation.

Reporting for specific materials, systems and methods

We require information from authors about some types of materials, experimental systems and methods used in many studies. Here, indicate whether each material, system or method listed is relevant to your study. If you are not sure if a list item applies to your research, read the appropriate section before selecting a response.

Materials & experimental systems

n/a	Involved in the study
☒	☐ Antibodies
☒	☐ Eukaryotic cell lines
☒	☐ Palaeontology and archaeology
☒	☐ Animals and other organisms
☒	☐ Human research participants
☒	☐ Clinical data
☒	☐ Dual use research of concern

Methods

n/a	Involved in the study
☒	☐ ChIP-seq
☒	☐ Flow cytometry
☒	☐ MRI-based neuroimaging
